# Professional nurses supporting learners during professional socialisation in Limpopo province

**DOI:** 10.4102/hsag.v29i0.2450

**Published:** 2024-01-25

**Authors:** Julia L. Mafumo, Takalani R. Luhallma, Maria S. Maputle

**Affiliations:** 1 Department of Advanced Nursing Science, Faculty of Health Sciences, University of Venda, Thohoyandou, South Africa

**Keywords:** clinical learning areas, learner nurses, professional nurse, role model, socialisation, support

## Abstract

**Background:**

Professional nurses play a significant role in the professional socialisation of learner nurses during clinical placement. Clinical placements are areas of experiential learning as learner nurses come in contact with real-life experiences. Professional nurses are custodians of learners and need to offer them support.

**Aim:**

The study sought to explore the role of professional nurses in supporting learner nurses during professional socialisation.

**Setting:**

Four clinical health facilities in Limpopo province at different levels of care were purposely sampled to obtain information from different levels of care.

**Methods:**

An ethnonursing approach and qualitative, explorative design was used. Non-probability purposive sampling was used to select 25 professional nurses. The criteria were professional nurses with 3 years of experience in professional socialisation and working in institutions accredited for clinical placement of learner nurses registered in the undergraduate programme. Data were collected through a face-to-face interview until data saturation was reached. Tesch’s open coding system was used to analyse data.

**Results:**

Professional nurses acknowledged that their responsibilities in supporting learners during professional socialisation are mentoring, teaching and being competent practitioners. Professional nurses were aware of their role in transferring ethical skills and knowledge to learners through professional socialisation.

**Conclusion:**

The effectiveness of professional socialisation of learner nurses depends on the support offered during clinical placements.

**Contribution:**

The study adds to the body of knowledge in nursing education and practice because when learners are offered support in the clinical areas, their learning journey will be of positive experience leading to competent practitioners.

## Introduction

Nursing education training comprises theory and practice components. After learner nurses are given the theory on the provision of care, they are allocated to the clinical learning areas for work-integrated learning (WIL). When learners are in the clinical learning areas, they are under the supervision and guidance of professional nurses as stated in the *Nursing Act 33 of 2005*. The supervisory role of professional nurses includes transferring knowledge, skills and ethical professional values through professional socialisation. Professional socialisation is described as a process by which the neophytes of the nursing profession acquire the specialised knowledge, skills, attitudes, values, norms and interests needed to perform their roles acceptably (Moradi et al. 2017). It is necessary that learner nurses be involved in the ward activities, so that learners can be able to integrate the theory from nursing education institutions and practice in the clinical learning areas (Adjei et al. 2018). Furthermore, professional socialisation can be described as the formation and internalisation of the characteristics that are typical of a profession starting from the commencement of training and continuing throughout the practice of an individual (Dinmohammadi, Peyrovi & Mehrdad 2017; Salisu et al. 2019). Professional socialisation has been indicated by De Swardt, Van Rensburg and Oosthuizen (2014) as the process of involving learners of a professional group getting immersed in the professional culture.

Professional socialisation is the basis for clinical practice, and it plays a significant role in the development of nursing identity. The aim of educating learner nurses is to ensure that students are equipped with skills to be able to render quality health services during their training period and beyond, when they are in practice. Quality nursing care is provided to clients, families and patients per the standard set by the profession. Supporting learner nurses during professional socialisation in the clinical learning areas provides the learner with the opportunity to observe good practice and can imitate the practice (Brown, Stevens & Kermode 2011). In a study conducted in the United Kingdom, Manchester by Jack, Hamshire and Chambers (2017), findings reveal that professional nurses have a role in supporting learners during professional socialisation. Their role was cited as that of being mentors through acting as exemplary role models and providing teaching and acting in an acceptable manner within the profession. Their view was that if professional nurses act in a manner that upholds the ethical and moral standards of the profession, it will promote good learning practice and improve the professional socialisation of learners in the clinical areas.

Professional socialisation is described as a process of metamorphosis where learner nurses are transformed from an outsider to the insider and the process is influenced by professional agents and conditions (Dinmohammadi et al. 2017). These agents are professional nurses in the clinical learning areas who support and guide learners. In their supportive role, professional nurses are also expected to create an environment that will promote a culture of learning and professional socialisation (Mafumo & Netshikweta 2022).

A South African study by De Swardt et al. (2014) revealed professional nurses’ views on the role in the professional socialisation of learners, as to be role models who should behave in a manner that is of good conduct. The authors further state that professional nurses indicated that in their role as supervisors, they need to be skilled and guide students to be as such so that in the end the graduates who complete the training are skilled and competent.

## Problem statement

The support of learner nurses is the cornerstone for professional socialisation in the clinical learning areas. When learners are allocated in clinical learning areas, they depend on skilled practitioners to show them the way and lead them. Often these learners complain about the poor support they receive from professional nurses and other staff. De Swardt, Van Rensburg and Oosthuizen (2017) concluded that in order to support learners during professional socialisation, professional nurses need to be role models in shaping their professional journey. The professional nurses in the wards have a teaching function whereby they are expected to offer such in order to assist learner nurses in the integration of theory and practice (Bastable 2021). Available literature (Bussard & Lawrence 2019; Hunter & Cook 2018) describes role modelling of professional nurses in professional socialisation of students, but not much is said about their inclusive role in professional socialisation of learner nurses. Learners often complain to say that professional nurses refuse to teach them saying it is the responsibility of lecturers and not professional nurses. The researcher as a nurse educator who follows up with learners during clinical accompaniment would find learners practicing unsupervised and idling in the clinical areas. This is an indication that the support of learners in the clinical areas is inadequate and hence the desire to explore the perception of professional nurses regarding their role in supporting learner nurses during professional socialisation.

## Theoretical framework

The study is based on the theoretical framework of Benner’s Novice to Expert Model (1984), which describes how learner nurses develop comprehension of patient care through theoretical training and experiential learning (Ozdemir 2019). The model has five stages. When learner nurses are in the clinical areas, they are under the supervision of professional nurses who offer support at different stages of learning. The novice stage is in the first year wherein nursing education institutions provide students with the theory of nursing, and simulations are carried out to expose learners to situations they will encounter in the clinical learning areas (Mafumo & Netshikweta 2022; Thomas & Kellgren 2017).

The second stage is when the learner nurses go to the clinical learning areas for the first time; at this stage, professional nurses support learners through direct supervision and guidance during the performance of allocated activities in order to integrate theory into practice (Ozdemir 2019). The third stage is the competent stage where learners gain competency in planning patient care. Professional nurses support learners through the evaluation of allocated responsibilities. Evaluation of performance is performed continuously so that learners keep track of their performance (Ozdemir 2019). The fourth stage is the proficient stage where learner nurses are more confident in the provision of patients’ care. The support by professional nurses is minimal as learners are less dependent on professional nurses. Professional nurses act as role models in the execution of tasks so that learners can emulate them. In the final stage of competency, learner nurses are more competent and confident in practice. Professional nurses support learners by allocating them to more senior tasks and guiding them in the role of professional nurses. Professional nurses act as mentors in the professional culture by reinforcing ethical values embedded in nursing practice.

### Purpose

The study sought to determine the role of professional nurses in socialisation of learner nurses during placements in the clinical learning areas of Limpopo province, South Africa.

### Objectives

The objectives were to describe the role of professional nurses in socialisation of learner nurses during clinical placements in the clinical learning areas of Limpopo province, South Africa, and to suggest recommendations to strengthen the professional socialisation of students during such clinical placement.

## Research methods and design

The study was a qualitative, ethnonursing research to describe the role of professional nurses during their interaction with learner nurses within the culture of nursing practice (Grooves, Burns & Gray 2013). The culture of nursing includes the values, moral standards and ethics of the profession. The design was to describe the role of professional nurses in the professional socialisation of learner nurses according to their own understanding.

### Setting

In this study, the setting was four sampled clinical healthcare facilities in Limpopo province, South Africa, where learner nurses registered for the undergraduate nursing programme are placed for clinical experience. The province has five districts, and the two districts sampled have the university and the college where learner nurses are trained. One district has eight hospitals wherein two are tertiary hospitals and six are district hospitals; the hospitals are accredited for students’ clinical practice. The other district has seven hospitals in which six are district hospitals and one is a regional hospital; however, five hospitals are accredited for students’ clinical practice. Limpopo province is a rural province located in the northern part of South Africa with a population of 5 million people. The most spoken languages are Sepedi, Tshivenda and Xitsonga.

### Sampling of institutions

Four healthcare facilities within the province where learner nurses training for the undergraduate nursing programme were allocated for clinical learning were purposively sampled as these were the areas of professional socialisation. The two districts out of five in the province were purposively sampled as they are the ones with the university and the college with registered learner nurses. The sampled hospitals were at different levels of care, tertiary, regional and district hospitals. This was performed to obtain information at those institutions, as the type of care might influence the responsibilities of professional nurses. The rationale for selecting these healthcare facilities stemmed from the prevalent reports by learners indicating inadequate support from professional nurses during clinical placement.

### Sampling of participants

Non-probability purposive sampling was used to sample professional nurses who were working at the sampled institutions and were responsible for the professional socialisation of learners. Professional nurses are custodians of professional socialisation in the clinical learning areas and the researcher assumed that they were the correct category of nurses to provide adequate information regarding their role in the professional socialisation of learners. A professional nurse is defined as a person who is qualified and competent to independently practice comprehensive nursing in the manner and to the level prescribed and who is capable of assuming responsibility and accountability for such practice (*Nursing Act 33 of 2005*). The inclusion criteria for professional nurses were that they should have been in the professional socialisation of learners for a period of not less than 3 years. Both male and female professional nurses were part of the study.

### Data collection

Data were collected over a period of 7 months, starting in May 2019 and lasting up to November 2019. The researcher visited each healthcare facility for several days to meet different staff as they were working in shifts. The researcher was the main research instrument who collected data. The researcher and the participants did not know each other prior to data collection to minimise bias and conflict of interest. Professional nurses who were interested in participation were recruited through the nursing service manager who gave permission to address them. The first meeting was arranged with the willing participants where the researcher introduced herself to the participants and the purpose and significance of the study were explained. Informed written consent was obtained before data collection. Data were collected through a one-on-one interview. The interviews were held at the venues provided by the institutions. The language used was English and vernacular to those who had limited understanding of English. The participants’ right to privacy was ensured whereby their names were protected and they were given alphabets. The researcher used an interview guide. The following questions were asked of the participants: (1) ‘as a professional nurse, what is your role in the professional socialisation of learner nurses?’ and (2) ‘what qualities are needed for a professional nurse to support learners during professional socialisation?’ The researcher went on to probe participants to give clarity on some points given. Descriptive field notes of factual data such as the date, time, setting and behaviours of participants during interviews were taken during data collection and were later used to support data analysis (Phillippi & Lauderdale 2018). Permission to use a voice recorder was sought from participants. Its use was to capture the narrative data so that the researcher could listen and compare them to the field notes after data collection. Participants responded well to the two questions as adequate information was provided and no follow-up interviews were necessary. Data were collected until saturation was reached at participant number 25; however, the researcher interviewed three more participants to ensure that data saturation was indeed reached. Each interview lasted for 45–55 min.

### Data analysis

Tesch’s (1990) open coding method was used to analyse data. During analysis, the concepts that belonged together were grouped to form themes and subthemes. Data analysis started during data collection when participants were responding to the asked questions as some of the common views shared by the participants were giving shape to the development of themes. The researcher transcribed the recorded data verbatim immediately after data collection when saturation was reached. The interviews that were carried out in the vernacular were translated by the language expert. All the transcripts and field notes were carefully read to gain a sense of the whole several times, to acquaint the researcher with the data collected and to jot down some ideas that came to mind; similar topics were clustered together. After data coding, data were reduced and examined closely for similarities and differences. Similar data were grouped into main themes, themes and subthemes. The two experienced researchers in qualitative research, one being an independent coder, were given the transcripts and field notes to analyse data independently; thereafter the researchers met and discussed the themes that were derived, and the consensus was reached.

### Measures to ensure trustworthiness

The researcher used Lincon and Guba’s (1985) four general criteria to ensure trustworthiness in qualitative research, which are credibility, transferability, dependability and confirmability. Trustworthiness refers to the degree of confidence qualitative researchers have on their data assessed (Polit & Beck 2017). Credibility is confidence in the truth of the data and collection thereof (Brink & Van der Walt 2006). To ensure credibility, the researcher used prolonged engagement where data were collected over a period of 7 months, thereby allowing more time spent with the participants. An in-depth understanding of the phenomenon of professional socialisation of learner nurses was obtained during engagement. In qualitative studies, triangulation is used to increase the credibility and validity of research findings; furthermore, it involves the examination of evidence from various sources, which is then used in the justification of themes (Noble & Heale 2019). Triangulation was ensured by the thorough sampling of the institutions and participants, taking field notes, observing the behaviour of the participants and using a voice recorder so that all the information from participants was recorded. After data collection, member checking was carried out when the researcher went to the professional nurses of one sampled institution to confirm if the findings were what the participants indicated. Transferability is the applicability of the study results to be applied to other populations (Prion & Adamson 2014). In the study, this was ensured through a proper sampling of the participants. To ensure transferability, the researcher thoroughly described the research process and the results of the study. The sampling of participants was purposively carried out to ensure that the participants met the criteria of the intended population. To ensure dependability, the researcher worked with two experienced qualitative researchers who assisted in data analysis and coding of themes. The researcher also ascertained confirmability by remaining focussed on conducting the study by following the research process during data collection, sampling, data analysis and interpretation of the findings.

### Ethical considerations

After obtaining ethical clearance, information was redacted to maintain the integrity of the review process. Letters requesting permission to collect data were sent to the responsible structures and institutions, and permission was granted. The ethical principles of anonymity, autonomy, beneficence and justice were explained and adhered to throughout the study. Participants who were willing to participate were made to sign a consent form.

The proposal was presented to the University of Venda Department and Faculty Research Committees. After approval it was presented to the University Higher Degree committee. It was then presented to the University of Venda Ethic Committee where ethical clearance was issued (SHS/19/PDC/1103).

## Results

Participants in the study were 18 females and 7 males. Ten of them had a qualification in general nursing (community, psychiatric) and midwifery, eight had a bachelor’s degree in nursing degree and seven had a diploma in nursing. Their ages ranged between 25 and 55 years. The population was heterogeneous. Eight participants were from a tertiary hospital, and seven were from regional hospital, and district hospitals had five participants each.

The findings revealed that professional nurses viewed their role of supporting learner nurses during professional socialisation to be important. Themes and subthemes from the findings are indicated in Table 1.

**TABLE 1:** Themes and subthemes that emerged from the findings.

Themes	Subthemes
Mentoring of learner nurses	The appearance of a professional nurse
	The conduct of the professional nurse
Providing teaching and learning	Promoting a positive learning environment
	Using teachable moments
Professional nurse as a practitioner	Being competent in practice
	Professional nurse as a clinical supervisor

### Theme 1: Mentoring of learner nurses

Findings in the study revealed that professional nurses consider their role to be mentors to learners. A mentor is defined as a relationship consisting of assessing, supervising, preceptor and coaching learners (Matza, Garon & Que-Lahoo 2018). As mentors, professional nurses are expected to guide and coach learners. In the first year of training when learners are still in the advanced beginner and competent stages of learning, they need direct mentoring and support from professional nurses. At this stage, learners need professionals who will guide them and support them. In their mentoring roles, professional nurses indicated the need to present themselves as professional as possible and behave in a manner that will uphold the moral and ethical codes of the profession.

#### Subtheme 1.1: The appearance of a professional nurse

The findings revealed that professional nurses viewed their appearance and the way they dress as influences on how learner nurses view the profession. The appearance of a professional nurse indicates the image of the profession. Regulation 1201 of the *Nursing Act (33 of 2005)* states how the uniform of the nurse should be worn. The uniform is not only the attire but also the inclusion of distinguishing devices. If the professional nurses put on the uniform as prescribed by the institution, learner nurses will be able to copy and feel that they also have the responsibility to put on the prescribed uniform. The professional image of nursing creates a context in which the public views nurses and the nursing profession. The professional image of a nurse is an emotional connection with a patient and the nurse and the care that the patient receives (Hatfield et al. 2013). To support the subtheme:

‘My perception of my role as a professional nurse in supporting learners during professional socialisation is that I must be a mentor to the learners. The professional nurse must always be in a complete full uniform with distinguishing devices so that I teach the learners how to dress properly. The hair style must also be acceptable not like where a person can have pink or purple coloured hair. If as the professional nurses I am like that, the students will see how I dress and want to be like me.’ (Participant H, Female, Hospital P)

Another participant indicated that:

‘My perception is that as a professional nurse I need to maintain the image of the profession by putting on a clean and neat uniform that makes me proud as a nurse in order to teach young nurses about the image of nursing.’ (Participant B, Female, Hospital D)

#### Subtheme 1.2: The conduct of the professional nurse

In this subtheme, professional nurses indicated that their behaviour and conduct might influence the professional socialisation of learners. The professional nurse indicated that their role was to behave in a manner that will uphold the moral and ethical codes of the profession. When learner nurses go to the clinical learning areas, they have no knowledge of the activities in the clinical areas, they only have the theoretical background from the nursing education institution, which they acquired during the novice stage of Benner’s stage of learning. They look up to professional nurses and whatever they do might be right in the eye of the learner. Nursing has its values and characteristics that everyone in the profession should adhere to (Niederriter, Eyth & Thoman 2017).

In support of the subtheme:

‘My role in supporting learners during professional socialisation is to be a mentor of learners. I must guide them to behave properly through my conduct. I must behave in such a way that it is not bad for learners to copy wrong things. I must speak to patients and staff politely in a respectable manner and not shout at people. Also, as a professional nurse I must always tell the truth and not lie. I must not steal patient food because if I do that, learners will be observing, and they must do the same tomorrow.’ (Participant C, Male, Hospital S)

‘In professional socialisation of learner nurses, my role in supporting them as a professional nurse is to show them to be empathetic and sympathetic towards patients. I should show them how to communicate with patients and behave in a professional manner. I have the responsibility to guide learners to treat patients with dignity even when they are under stress as patients are human too.’ (Participant X, Female, Hospital T)

### Theme 2: Providing teaching and learning

An environment that is relaxed and has healthy communication between staff and learners will promote learning, which is a significant component of professional socialisation. Clinical areas are strenuous and stressful by nature, so professional nurses have a role to create a positive clinical learning environment where learning and professional socialisation can effectively take place. Sometimes these learner nurses struggle to find appropriate ways where they can fit, especially in the clinical learning areas as they need to maintain the balance between the prevailing values from the university and college (Ewertsson et al. 2017). Hence, professional nurses should come in to support the learners from the beginning to the expert stage of learning so that they can find their feet and help them to be properly socialised into the profession and respect the ethics and morals of the profession. The journey to professional socialisation is not a smooth one where one must suddenly adopt and adapts to a certain way of behaviour and conduct making. The people who can help hold the hand of learner nurses in this journey are professional nurses as they are the custodians of teaching and learning in the clinical learning areas.

#### Subtheme 2.1: Promoting a positive learning environment

Professional nurses indicated their willingness to support learners by promoting a positive clinical learning environment where learners could feel free to learn. Clinical learning environment could be stressful to learners, especially during the first placements. According to Burner’s model (1984), learners in the advanced beginner stage, which is in the first year of study, are the most stressed as they do not know what is expected of them in the clinical areas; it is at this stage where the support of professional nurses is needed the most:

‘During the first year of clinical placement, learners are often shy and scared and not free. They also don’t know what to do or what to ask. In my role, I ask the students about what is that they want to learn, and when they tell me I make an appointment to teach them. I also ask about how they are coping, whether they have any challenges and even ask about their well-being. In so doing I think I am giving them support to feel welcome.’ (Participant E, Male, Hospital T)

‘As a professional nurse, I support learner nurses in ensuring that they feel welcomed when they are in the clinical learning areas. Some of them have not been to the hospital before and are scared so, I make them feel welcomed and tell them to be free and ask questions so that we can help them. We even make jokes so that they don’t feel stressed seeing sick patients.’ (Participant D, Female, Hospital D)

#### Subtheme 2.2: Using teachable moments

The findings of the study revealed that professional nurses are aware of their teaching responsibility and were willing to execute the responsibility. Professional nurses felt that they need to teach learners, especially about the conditions that are found in the particular unit. They stated that during the provision of care, they engage learners by explaining step by step the procedure that they are doing especially those that are not common. When learners go to the clinical learning areas, they are in the advanced stage of Benner’s and have the theoretical background and depend on professional nurses to assist in putting theory into practice. Professional nurses accepted the teaching of learners in the clinical areas as their responsibility and indicated the significance of teaching to learners. The following transcripts support the theme:

‘My role in supporting learners during professional socialisation is to teach them when they are in the clinical learning areas. In our ward, we have a teaching program where we offer teaching three times a week on the common conditions that are in the ward. We even delegate learners to teach. Also, during the performance of procedures, I usually want the student to be nearby so that I teach them what I am doing and give them the opportunity to ask questions.’ (Participant L, Female, Hospital S)

‘As a professional nurse, my role in supporting learners is to provide learners with teaching about the common conditions in the ward. In most cases, these students have not seen the different conditions. This offers them support as they gain insight into the ward activities and gives them confidence. When they come to the ward, I teach them about those conditions.’ (Participant M, Female, Hospital P)

### Theme 3: Professional nurse as a practitioner

Professional nurses view their role as being competent in practice and being the person who ensures that learning takes place through clinical supervision. They indicated that for them to be adequately socialised learners professionally, they need to be competent and ensure that learner needs are attended to.

#### Subtheme 3.1: Being competent in practice

The findings revealed that professionals value the significance of competency in practice to be their role in the professional socialisation of learners. They indicated that they need to be competent in problem-solving, communication, ward administration and provision of care. The findings were supported by Lamont, Brunero and Woods (2015) who indicate that competency is one of many factors that influence positive clinical placement, which is important in the professional socialisation of learners. The scope of practice R786 of the *Nursing Act* (*33 of 2005*) indicates that the professional nurse is expected to be competent in the provision of care to patients. Professional nurses guide students in the competent, proficient stages to be experts in the performance of their duties so that they become competent practitioners. Competency starts during training through professional socialisation and continues throughout the practicing career of an individual. Competency is associated with patient safety so professional nurses need to be competent so that learner nurses under their supervision and professional socialisation can be competent. The issue of competency was stressed by many:

‘The role that I play as the professional nurse in supporting the professional socialisation of learners is that I must be competent in whatever I do in the clinical learning environment. In the ward, there are many activities that are done that I am involved with on daily basis. I can’t be with students and do wrong things in front of the students. This will lead to students witnessing wrong things and will not know the correct things.’(Participant S, Female, Hospital D)

In addition, participants:

‘In order to support learner nurses, I need to ensure that the learners are competent in their practice. They can only be competent if they see me practicing with competency. My role is to do procedures with diligence and competency so that learner nurses observe and emulate the right things. I also guide learner nurses when they are performing procedures so that they do them correctly.’ (Participants P, Male, Hospital T)

‘My role in supporting learners during professional socialisation of learners is to teach learners how to do the procedures in the ward. In order to do that, I need to be competent so that I teach them to do the right things. As a professional nurse competency for me is very important in the professional socialisation of learners.’ (Participant W, Female, Hospital P)

#### Subtheme 3.2: Professional nurse as a clinical supervisor

Findings indicated that professional nurses view clinical supervision as their role in professional supervision. Professional nurses are people who are always working hand in hand with students, so they need to oversee that the training needs of learners are met. In their supervisory role, professional nurses support learners whereby those who are in the early stages of learning will need constant direct supervision, and senior students in the competency, proficient and expert stages will need indirect supervision:

‘My role in supporting professional nurses is to supervise learners when they are in my unit. I need to ensure that they meet their learning objectives which are provided by the university. I check their procedure files s that they are complete before they finish their clinical placement.’ (Participant Z, Male, Hospital T)

‘As a professional nurse, my role is to supervise learners. Learner need someone who takes care of their learning needs. I constantly check on their performance and when I notice that they have challenges, I make time to show them what should be done. Sometimes learners hide behind others and during clinical supervision, I can identify the weak ones and assist them with practical skills. Though at times it becomes difficult to supervise learners due to overcrowded and busy wards, I still consider clinical supervision as my role in the professional socialisation of learners.’ (Participant L, Female, Hospital S)

## Discussions

The study aimed to determine the role of professional nurses in supporting learners during professional socialisation in the clinical learning areas. Learners depend on professional nurse to support and supervise them throughout their training as they develop and find themselves in the profession. The foundation of practical placement is the theory provided by the nursing education institutions in the novice stage of Benner’s model whereby students are equipped with the knowledge and prepared for situations that they will encounter in the clinical learning areas (Ozdemir 2019). Starting in the second stage of the model, professional nurses who are with the learners most of the time in the clinical areas take the centre stage in supporting learners during clinical practice. The professional nurse’s role in supporting the professional socialisation of the learner does not only help the student to succeed in their training but also in their personal and professional journey to become a competent and skillful practitioner one who is caring and compassionate, who cares for people in a humanising way and one who has the resilience to challenge those who are not demonstrating humanised care (White et al. 2018). Professional nurses can mentor learners through role modelling (Setati & Nkosi 2017). Mentoring of learner nurses is seen as guiding and offering support to learners, which influences professional socialisation during clinical placement. Professional socialisation and development of learners depend on successful mentoring relationships where learners are taken under the wings of professional nurses. In the first year of training when learners are in the advanced beginner and competent stage, they need to be closely mentored as they are in the early stages of learning and do not as yet possess the necessary skills.

Mentorship as a tool for professional socialisation has benefits, which include but are not limited to job satisfaction, performance and commitment, and career success for learner nurses (Navarra et al. 2018). In another study by Mendes, Da Cruz and Angelo (2015), students perceived that their instructors possessed both positive attitudes and a caring demeanour, which is a good basis for mentoring of learners. The appearance of the professional nurse influences the image of the profession. The public opinion is that nurses should dress professionally, especially during their encounters with patients. In the nursing profession, the wearing of a uniform is further viewed as an extension of professionalism (Appiah, Agyeiwaa & Amponsah 2018; Daigle 2018). The sentiment was further shared by Pullen and Alley (2015) who stated that appearance can create a positive or negative appearance of a nurse whereby a dirty wrinkled uniform has poor posture and decreases the credibility of the nurse to the patient. Therefore, professional nurses need to be in full uniform to uphold the image of the profession, which is part of professional socialisation. Wills et al. (2018) shared the sentiments through their study, which indicated that a person with a good professional image is portrayed as more professional, and this conduct influences the professional socialisation of learners. As professional nurses act as mentors to learners, how they dress up in their uniform might influence the way the learner turns out to dress in the near future.

As argued by Setati and Nkosi (2017), mentoring can be achieved through role modelling of professional values and conduct. If professional nurses in the clinical learning areas are practicing the right things, this could be internalised by the learner nurses who will reproduce the behaviour when in practice. Professional nurses and clinicians are viewed as strong role models by learners. It is through role modelling that professional nurses can mentor learner nurses and consciously or otherwise transfer professional skills and values. As role models, professional nurses need to demonstrate therapeutic communication, critical thinking, compassion, enthusiasm and positive attitudes all the time in the clinical areas. This is supported by Gibbs and Kulig (2017) indicating that learners describe that the clinical instructor, in this case the professional nurse, was an important factor in shaping their ability to think critically. De Swardt et al. (2017) in their studies on supporting students in professional socialisation concluded that students consider nurses in the clinical field the most influential role models in shaping their clinical practice, consequently their socialisation process. The authors further revealed that professional nurses in the clinical area act as role models through the demonstration of good communication skills, positive attitudes and caring. Professional nurses’ role in professional socialisation comprises the responsibility to be able to instil professional values to learner nurses through role modelling. The sentiment was shared by De Swardt et al. (2017) who alluded that a good work ethic is important in nursing practice as it influences the learner nurses’ experience regarding the identification and handling of ethical problems.

Professional nurses as clinical supervisors have a significant role in supporting learner nurses’ professional socialisation and development. The teaching responsibility of professional nurses plays a significant role in the professional socialisation of learner nurses. When learner nurses go for clinical experience, they only have the theory from the nursing education institution and depend on the professional nurses to integrate theory into practice (Adjei et al. 2018). Through formal and informal teachings that are provided in the clinical learning areas, learner nurses are given the opportunity to understand and know the conditions of patients better than through imagination.

The role of professional nurses towards learners is further affirmed by the nursing regulatory body. The professional nurse has the responsibility to supervise learner nurses. The *Nursing Act* (*33, 2005*) indicates that the responsibilities of the professional nurse in the clinical area include clinical supervision of learners. When learner nurses are in the clinical learning areas, they need to be assisted to integrate the theory acquired at the NEIs with the activities in the clinical learning environment. A study in Australia by Anderson, Moxham and Broadbent (2018) revealed that professional nurses have insight into their teaching function to learner nurses. The authors further indicated that professional nurses believed that teaching students is the right thing to do and they have the responsibility to ensure that it happens. Creating a supportive clinical learning environment is enormously important in ensuring positive professional socialisation. The clinical learning areas are challenging in nature and an environment that is relaxed and welcoming can make it easy for learner nurses.

In the proficient stage, students are more competent in practice and they practice with confidence and with minor supervision from professional nurses. Competency in nursing practice plays a huge role in transferring skills and professional socialisation of learners. Learning through observation has an impact on professional socialisation as learner nurses internalise what they observe. Competent professional nurses will be able to transfer the skills to the learners through professional socialisation. Society expects nurses to be competent as competency leads to an improved quality of patient care and patient satisfaction (Karami, Farokhzadian & Foroughameri 2017).

A study by Phillips et al. (2017) alluded that students prefer to be allocated in a more positive, supportive clinical learning environment as they view the environment as contributing positively to learning and professional socialisation. Several studies supported the fact that learner nurses often have a high level of stress compared with students from other disciplines such as science and law, so learning under stress can be very disturbing and might make it difficult to learn (Kupcewicz et al. 2020; McCarthy et al. 2018; Valero-Chillerón et al. 2019). Stress is common during the first stages of advanced beginner and competent in Benner’s stages of learning as learners are not sure of what to do or are intimidated by the nature of the clinical learning environment. If the stress in the clinical learning area is not effectively handled, it might affect professional identity and socialisation wherein learners will fail to correctly fit into the profession (Graham et al. 2016). McCarthy et al. (2018) cited that some of the intrinsic forecasters of stress in students could be attributed to self-control and self-efficacy, coping styles, personality factors and mental health issues. Professional nurses need to support learners so that they allay the anxiety and reduce the stress that learner nurses might have.

## Limitations

Even though the researcher made efforts to ensure the trustworthiness of the study, qualitative studies often have challenges as they indicate the perception of one group of participants. The study was conducted in rural areas and the limitation could be that if the study was in urban areas, it might have yielded different results because of the fact that in rural areas the clinical learning areas are small with fewer staff and patients than in urban areas. The study concentrated on learner nurses in undergraduate studies and support for postgraduate professional socialisation might yield different results as these students had undergone previous training and most of them are professional nurses.

## Recommendations

The results of the study were shared with the managers and professional nurses in the sampled institution. Nursing practice as the backbone of the healthcare system needs practitioners who perform their tasks with diligence and competency. These attributes are transferred to the new recruits in the profession by the qualified staff through support.

### Recommendations for nursing practice

It is therefore recommended that professional nurses in the clinical learning areas act as mentors to learners at different levels of training. Learners who are still in the beginner and advanced beginner stages of Benner’s model need more constant mentoring than those in the other stages who need limited support. Learners aspire to be professionals and therefore the possibility of them copying what professional nurses do is higher; it is therefore recommended that whatever professional nurses do, they should act as exemplary role models to the learners. Professional nurses need to create an environment conducive to learning through open communication and involving learner nurses in the decisions in the units.

Professional nurses should also be competent in practice. The employer should encourage self-development through private studies and offer job training to improve competency in staff. Professional nurses are to be kept up-to-date regarding current issues in healthcare.

### Recommendations for nursing education

Nurse educators from nursing education institutions should have a regular schedule to go to the clinical learning areas so that the challenges the students have can be attended to. Nursing education institutions should offer workshops to professional nurses to make them aware of their supportive and teaching responsibilities to learners.

### Recommendations for research

Clinical learning areas are crucial for learner nurses’ training. It is imperative to have research that will look into factors that hinder or make it difficult for professional nurses to nurture and support students during professional socialisation. As the results indicated, professional nurses have insight into their responsibilities.

## Conclusion

Professional nurses are aware of their roles in supporting learners during professional socialisation as mentors in the professional socialisation of learners. Their role in the professional socialisation of learner nurses is to be mentors who should act as exemplary role models in conduct and appearance as this affects the image of the profession. Professional nurses also indicated the importance of providing support to learners so that the learners are free to learn and adapt to the clinical learning areas. The significance of creating an environment conducive to learning and communication was emphasised. The issue of professional nurses as competent practitioners was discussed wherein the professional nurse who was competent was considered to be able to teach learners how to do things correctly and protect the patient from harm. Professional nurses also indicated that in their role modelling duties, they needed to protect the right of the patient from harm and abuse so that learners know how to protect the patients.
